# Decreased Intrinsic Functional Connectivity of the Salience Network in Drug-Naïve Patients With Obsessive-Compulsive Disorder

**DOI:** 10.3389/fnins.2018.00889

**Published:** 2018-11-28

**Authors:** Yun-Hui Chen, Su-Fang Li, Dan Lv, Gui-Dong Zhu, Yu-Hua Wang, Xin Meng, Qiang Hu, Cheng-Chong Li, Liang-Tang Zhang, Xiang-Ping Chu, Xiao-Ping Wang, Ping Li

**Affiliations:** ^1^Department of Psychiatry, Qiqihar Medical University, Qiqihar, China; ^2^Department of Psychiatry and Behavioral Sciences, Johns Hopkins University School of Medicine, Baltimore, MD, United States; ^3^Lishui Second People’s Hospital, Lishui, China; ^4^Department of Radiology, The Third Affiliated Hospital of Qiqihar Medical University, Qiqihar, China; ^5^Department of Clinical Psychology, Qiqihar Mental Health Center, Qiqihar, China; ^6^Department of Psychiatry, National Clinical Research Center on Mental Disorders, National Technology Institute on Mental Disorders, The Second Xiangya Hospital of Central South University, Changsha, China

**Keywords:** obsessive-compulsive disorder, salience network, resting-state, functional magnetic resonance imaging, intrinsic functional connectivity

## Abstract

Obsessive-compulsive disorder (OCD) patients have difficulty in switching between obsessive thought and compulsive behavior, which may be related to the dysfunction of the salience network (SN). However, little is known about the changes in intra- and inter- intrinsic functional connectivity (iFC) of the SN in patients with OCD. In this study, we parceled the SN into 19 subregions and investigated iFC changes for each of these subregions in 40 drug-naïve patients with OCD and 40 healthy controls (HCs) using seed-based functional connectivity resting-state functional magnetic resonance imaging (rs-fMRI). We found that patients with OCD exhibited decreased iFC strength between subregions of the SN, as well as decreased inter-network connectivity between SN and DMN, and ECN. These findings highlight a specific alteration in iFC patterns associated with SN in patients with OCD and provide new insights into the dysfunctional brain organization of the SN in patients with OCD.

## Introduction

Obsessive-compulsive disorder (OCD) is a psychiatric disorder characterized by two symptoms: intrusive, recurrent, distressing thoughts (obsessions) and/or repetitive behaviors (compulsions), with a lifetime prevalence of 2–3% (Ruscio et al., 2010). Although the pathophysiology of OCD remains unclear, neuroimaging studies have provided important insights into the neurobiological models of OCD. Many structural and functional magnetic resonance imaging (fMRI) studies reported the abnormalities in several cortical and subcortical regions including the orbitofrontal cortex (OFC), anterior cingulate cortex (ACC), striatum, and thalamus, which are part of the pathophysiological model of cortico-striato-thalamo-cortical (CSTC) circuitry for OCD (Menzies et al., 2008; Harrison et al., 2009; Del Casale et al., 2011). The salience network (SN), composed of dorsal anterior cingulate, anterior insular cortices and several subcortical brain areas has been shown deteriorated connectivity with CSTC circuit activity in patients with OCD, which suggest that SN may also be involved the broader pathophysiology of obsessive-compulsive phenomena (Harrison et al., 2013; Zhu et al., 2016).

The recently proposed “triple-network” model emphasized the aberrant intrinsic functional connectivity (iFC) patterns within and between the default mode network (DMN), executive control network (ECN), and SN as core features of psychiatric disorders (Menon, 2011). Altered iFC within and between the DMN, ECN, and SN have been reported in patients with OCD (Stern et al., 2012; Posner et al., 2016; Fan et al., 2017a). As a core brain network, the SN is involved in detecting and filtering internal and external salient information (Sridharan et al., 2008; Menon, 2011). In addition to the intra-network function, SN also plays an important role in monitoring interactions between ECN (task-positive network) and DMN (task-negative network). It is thought the SN initiates transient control signals that engage the ECN to mediate cognitive control processes while disengaging the DMN when a salient external stimulus is detected (Menon, 2011; Fan et al., 2017b). Patients with OCD have difficulty in switching between obsessive thought and/or compulsive behavior, which may be related to a dysfunction of SN in engaging task-positive ECN and disengaging task-negative DMN (Gürsel et al., 2018). IFC analyses within and between brain networks have shown to provide important insights into the neural deficits of psychiatric disorders (Shin et al., 2014). However, little is known about the changes in intra- and inter- iFC of the SN in patients with OCD.

Previous resting-state fMRI studies have indicated abnormal iFC within and between SN and other network. However, the results have somewhat been inconsistent. For example, within the SN, Fan et al. (2017a) demonstrated that patients with OCD exhibited greater iFC in the bilateral ACC within the SN using independent component analysis (ICA), and they also found elevated right insula-left dorsal ACC connectivity within the SN in patients with OCD with preserved insight into their symptoms (Fan et al., 2017b). For inter-network connectivity with SN, Posner et al. (2016) and Wang et al. (2018) both found increased iFC between the SN and the DMN in patients with OCD, as well as between the SN and ECN (Fan et al., 2017a). However, other researches revealed decreased iFC between SN and ECN and extending to DMN in patients with OCD (Stern et al., 2012; Gürsel et al., 2018). The differences among these results in OCD may, at least in part, be attributed to different methods and parameters used for seed definition (Stern et al., 2012; Posner et al., 2016). Furthermore, the functional connectivity based on assumptive and different seed definitions may lead to different results patterns, and limited in exploring the functional connectivity of possible sub-networks within a larger brain network (Stern et al., 2012; Posner et al., 2016). Alternatively, model- and seed-free approach such as ICA does not allow for exploration of relationships among subregions within a brain network (Fan et al., 2017a). Thus, in the current study, we systematically investigated the whole-brain iFC changes by first parceling the SN into 19 subregions according to publicly available atlas, then performed seed-based functional connectivity analyses using each of the 19 subregion as seed region. This method allows us to investigate the iFC between subregions within the SN as well as between SN and other parts of the brain. Furthermore, by correlating the changes of iFC with the severity of clinical symptoms in patients with OCD, it can help to elucidate brain–behavior relationships.

In present research, we aim to compare the iFC changes between all SN subregions and whole brain voxels in drug-naïve patients with OCD and healthy controls (HCs) using resting-state fMRI. Changes in iFC strength within SN and between the SN and other functional network was investigated, based on previous findings, it was hypothesized that the OCD group would exhibit abnormal iFC strength within the subregions of the SN, and between the SN subregions and another brain network. We also hypothesized that these changes would correlate with the clinical symptom of OCD.

## Materials and Methods

### Participants

Forty-three medication-free patients with OCD were recruited from outpatient and inpatient clinics at the Qiqihar Mental Health Center and the Fourth Affiliated Hospital of Qiqihar Medical University, Heilongjiang, China. Diagnoses were established using the Structured Clinical Interview for DMS-IV. The severity of OCD, depressive and anxiety symptoms were assessed with the Yale-Brown Obsessive Compulsive Scale (Y-BOCS), the 17-item Hamilton Rating Scale for Depression (HAMD) and the Hamilton Anxiety Rating Scale (HAMA), respectively. Only patients with a total score of 16 or higher on the Y-BOCS and a score less than 18 on the HAMD were included in the present study (Gottlich et al., 2015; Yang et al., 2015). All patients fulfilled the criteria of OCD, were right-handed and 18–60 years old. Exclusion criteria were the presence of neurological and other major psychiatric disorders other than OCD. At the time of the study, all patients with OCD had not taken any kind of psychotropic medication for at least 4 weeks. Fourteen patients with OCD did have a history of antiobsessive or antidepressant medication, such as selective serotonin reuptake inhibitors (SSRIs), serotonin and norepinephrine reuptake inhibitors (SNRIs) and clomipramine, eight patients had a history of antipsychotic medication, eighteen patients were drug-naïve. In addition, forty matched HCs were recruited using the Structured Clinical Interview for DSM-IV Axis I Disorders-Non-patient Edition. None of the HC subjects reported any history of neurological and psychiatric disorders.

This study was approved by the Research Ethics Committee at Qiqihar Medical University. All participants provided written informed consent.

### Image Acquisition and Preprocessing

RS-fMRI images were acquired with a 3.0-Tesla GE 750 Signa-HDX scanner (General Electric Healthcare, Waukesha, WI, United States) at the Third Affiliated Hospital of Qiqihar Medical University, Heilongjiang, China. Subjects were instructed to relax and lay as still as possible with their eyes closed, without falling asleep or thinking of anything in particular. The RS-fMRI scans were obtained using an echo-planar imaging (EPI) sequence with the following parameters: 33 axial slices, TR = 2000 ms, TE = 30 ms, FA = 90°, thickness/gap = 3.5/0.6 mm, FOV = 200 × 200 mm, in-plane resolution = 64 × 64. A total of 240 volumes were collected (8 min). None of the participants exhibited any clinically significant structural abnormalities upon visual inspection by two independent radiologists.

Resting-state functional images were analyzed using Data Processing & Analysis for Brain Imaging (DPABI) software (Yan et al., 2016). The first 10 volumes were discarded to ensure scanner equilibration. Preprocessing procedure included slice timing and motion correction, which then followed by normalization to a standard echo-planar image template in MNI space and resampled to isotropic voxel size of 3 mm. The resulting images were then smoothed with a 4-mm full-width half-maximum Gaussian kernel, linearly detrended, band pass filtered at 0.01–0.08 Hz, and scrubbed with a framewise displacement (FD) measure (with a threshold of 0.5 together with one preceding and two subsequent volumes) (Power et al., 2012; Liu et al., 2015; Han et al., 2016). Three patients with OCD were excluded due to more than 33% of the volumes were removed. The fMRI data of the remaining 40 patients have conducted the iFC analysis. The nuisance covariates, including 24 head motion parameters, white matter time course, and cerebrospinal fluid time course were modeled and regressed out using general linear model. We didn’t regress out the global mean time course, because doing so may cause artificial negative correlations in iFC analysis (Nalci et al., 2017). We calculated the mean FD for each participant, and there was no difference between patients with OCD and HCs (Table 1).

**Table 1:** Demography and clinical characteristics in patients with OCD and HCs.

	OCD patients (*n* = 40)	HCs (*n* = 40)	*X*^2^/*t*	*p*
Age (years)	27.28 8.16	27.00 8.25	0.15	0.88
Sex (male/female)	27/13	27/13	0.00	1.00
Education (years)	13.40 2.87	13.78 2.97	-0.57	0.57
Illness duration (months)	66.68 75.54			
Y-BOCS total score	24.90 5.73	1.10 0.87	25.96	0.00
Y-BOCS obsessive thinking	12.85 4.25	0.38 0.49	18.43	0.00
Y-BOCS compulsive behavior	12.05 4.62	0.70 0.72	15.36	0.00
HAMD	8.05 4.40	1.38 0.98	9.36	0.00
HAMA	10.83 6.55	1.20 0.99	9.19	0.00
FD	0.097 0.050	0.105 0.075	-0.54	0.59
Time points scrubbed out	1.13 2.256	1.00 2.418	0.25	0.95

*Data are presented as mean ± standard deviation or number or frequency. Y-BOCS, Yale-Brown Obsessive-Compulsive Scale; HAMD, 17-item Hamilton Depression Rating Scale; HAMA, Hamilton Anxiety Rating Scale; FD, framewise displacement. Variables of age, education, Y-BOCS total score, subscales score, HAMD score, HAMA score and FD were tested by two sample *t*-test, the results were indicated by *t*. Categorical data such as gender was tested using chi-squared tests, the results were indicated by *X*^*2*^*.

### Analysis on Functional Connectivity

The SN was identified with a publicly available atlas of functionally defined regions of interests (ROIs), developed by the Functional Imaging in Neuropsychiatric Disorders (FIND) lab at Stanford University^1^. This includes 19 subregions, from anterior region 1 (A1) to anterior region 7 (A7) and posterior region 1 (P1) to posterior region 12 (P 12), mainly including the medial frontal gyrus (medial FG), insula, dorsal ACC (dACC), middle cingulate cortex (MCC), the parietal cortex, and the cerebellum regions (see Supplementary Table S1 and Supplementary Figure S1).

Nineteen subregions of the SN were used as ROIs to calculate the iFC analysis between each seed region and all voxels in the whole brain using DPABI to examine whether the functional connectivity of the SN was altered in OCD. The mean time series was obtained and correlated with the time series of all the voxels in the whole brain. This results in 19 functional connectivity maps separately for each group. The correlation coefficients were transformed to standard *z*-values to achieve normality using Fisher’s r-to-z transformation. Two-sample *t*-tests were used to identify any brain regions that showed a significant iFC difference between patients with OCD and HCs. Bonferroni corrections were used for multiple comparisons. Given the number of seeds used, the corrected *p*-value was set at *p* < 0.05/19 = 0.00263 using the Gaussian random field (GRF) method (a voxel *p*-value < 0.001 and a cluster *p*-value < 0.00263).

The DMN, the ECN, and SN templates identified by the FIND lab were used to examine whether the iFC results belong to a specific brain networks. The principal regions involved in the DMN are the medial prefrontal cortex, ACC, posterior cingulate cortex (PCC)/precuneus, parietal cortex, and medial temporal regions (i.e., hippocampal and parahippocampal gyri) (Li et al., 2017). The ECN mainly included the parietal cortex, the dorsolateral prefrontal cortex (DLPFC), the angular gyrus, and the cerebellum region (Krmpotich et al., 2013) (see Supplementary Figure S2). The images were visualized with BrainNet Viewer (Xia et al., 2013).

To test whether iFC differences were correlated with the clinical presentation in patients with OCD, we correlated the connectivity strength within these areas that showed significant group differences with measures of Y-BOCS score, obsessive thinking score, and compulsive behavior score, respectively. HAMD score, HAMA score and FD values were included as nuisance covariates. We used a Bonferroni corrected threshold of *p* < 0.05/3 × 7 (0.002) to control for multiple comparisons.

## Results

### Clinical Characteristics

Clinical characteristics of patients with OCD and HCs were displayed in Table 1. There was no significant difference between the OCD and HCs groups in age, gender, education or FD values (all *p* > 0.05). There were significant group differences of total scores in Y-BOCS, HAMD, and HAMA subscales.

### Functional Connectivity Within the SN

**FIGURE 1:**
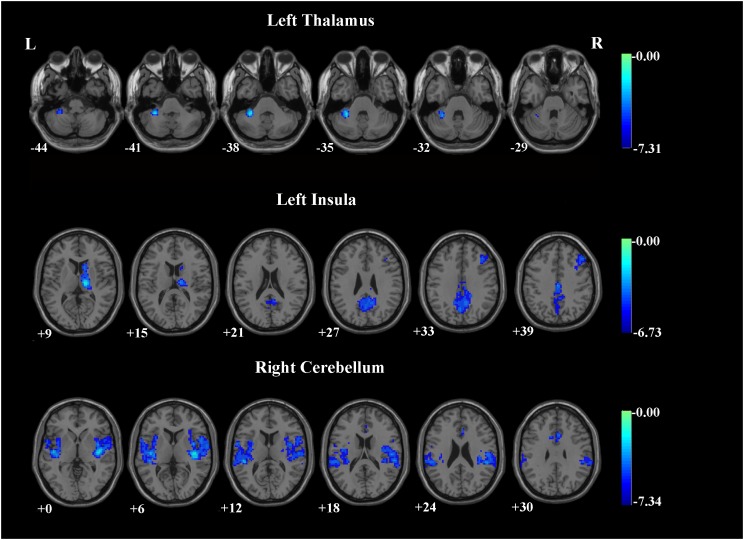
Brain regions demonstrating group differences of the iFC between SN subregions and whole brain voxels in patients with OCD. The threshold was set at a voxel *p*-value < 0.001 and a cluster *p*-value < 0.00263, two-tailed (Bonferroni corrected using the GRF method). L, left side; R, right side.

**FIGURE 2:**
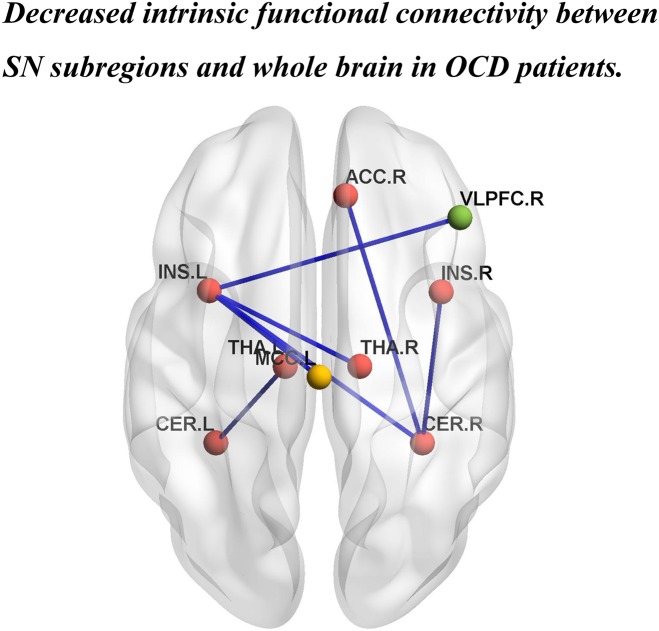
Brain regions showing significantly decreased iFC strength within the SN, and between the SN subregions and the DMN, and the ECN in patients with OCD. INS.L, left insula; INS.R, right insula; THA.L, left thalamus; THA.R, right thalamus; CER.L,left cerebellum; CER.R, right cerebellum. ACC.R, right anterior cingulate cortex; MCC.L, left middle cingulate cortex; VLPFC.R, right ventral lateral prefrontal cortex. The red, yellow, and green balls represent the SN, DMN, and ECN, respectively. The blue lines indicate decreased iFC strength in patients with OCD.

Patients with OCD exhibited significantly decreased iFC strength within the SN subregions compared to the HCs group (Table 2 and Figures 1, 2). Compared to HCs, patients with OCD exhibited decreased iFC strength between the left thalamus and the left cerebellum, between the left insula and the right thalamus, between the right cerebellum and the bilateral insula, and the right ACC.

**Table 2:** Brain regions demonstrating group differences of the iFC between SN subregions and whole brain voxels in patients with OCD.

SN	Side	Brain region	Brodmann area	Coordinates			Number of voxels	*t*
				*X*	*y*	*z*		
P7(L thalamus)	L	Cerebellum		-33	-42	-38	90	-7.31
P9 (L insula)	R	Thalamus		15	-18	9	258	-6.73
	L	MCC	23	0	-21	39	559	-5.34
	R	VLPFC	45	45	30	36	180	-4.70
P11(R cerebellum_6)	L	Insula	48	-40	-15	3	1325	-7.34
	R	Insula	48	40	-18	0	1673	-7.15
	R	ACC	24	6	18	30	287	-5.00

*SN, salience network; MCC, middle cingulate cortex; VLPFC, ventral lateral prefrontal cortex; ACC, anterior cingulate cortex. The threshold was set at a voxel *p*-value < 0.001 and a cluster *p*-value < 0.00263, two-tailed (Bonferroni corrections using the GRF method)*.

### Functional Connectivity Between the SN and Other Networks

Patients with OCD exhibited decreased iFC strength between the SN and the DMN compared to the HCs, which mainly found in the SN subregions (left insula) and the MCC (Table 2 and Figures 1, 2).

The iFC strength between the SN and the ECN was also significantly decreased in patients with OCD as compared to the HCs. Specifically between the SN subregions (left insula) and ventral lateral prefrontal cortex (VLPFC) (Table 2 and Figures 1, 2).

To exclude the effect of head motion, we did a correlation analysis between mean FD and FC values of regions showing significant difference between two groups, and found that there were no significant correlation between the mean FD and the FC values [all *p* > 0.05/1 × 7 (0.007) Bonferroni corrected]. Based on these results, we preliminary speculate that head motion may not affect the FC values of these regions.

### Relation Between Altered iFC Strength and Clinical Symptoms

The altered iFC strength within SN and between the SN and other functional network have no correlation with the clinical symptoms in patients with OCD (all *p* > 0.002).

## Discussion

The present study firstly split the SN into 19 subregions using the publicly available atlas and investigated the resting-state functional connectivity differences for each of the 19 SN subregions in drug-naïve patients with OCD vs. HCs. Consistent with our hypothesis, our results revealed significantly reduced iFC strength within the SN subregions in the OCD group compared with the HCs group. In addition to abnormalities within the SN, the OCD group also exhibited reduced iFC strength between components of the SN and the brain regions within DMN and ECN. These results provide evidence of a reduced connectivity within SN subregions, and between SN and DMN, and ECN. Consequently, these findings point to a specific alteration in iFC patterns associated with SN in patients with OCD.

Consistent with the results of a meta-analysis (Gürsel et al., 2018), decreased iFC strength within the SN subregions in the OCD was revealed in the present research, specifically in the bilateral insula, thalamus, and cerebellum. As a core component of the SN, insula plays an important role in information integration. Insula receives and integrates the internal and external stimuli to update expectations or to initiate actions (Menon and Uddin, 2010; Palaniyappan and Liddle, 2011). Decreased iFC strength between bilateral insula and thalamus, as well as cerebellum, may suggest a dysfunction in integration between these brain regions (Zhang et al., 2011). On the one hand, the thalamus and the cerebellum are unable to perform the task assigned by the insula; on the other hand, the insula also are unable to receive and integrate the information coming from the thalamus and the cerebellum. Therefore, the decreased intra-SN iFC may lead to the dysfunctions of SN in patients with OCD.

Another important finding in the present study is that patients with OCD exhibited significantly decreased iFC strength between the SN and the DMN (particularly between SN subregions and MCC) as well as decreased iFC strength between the SN and the ECN (particularly between SN subregions and VLPFC). The major functions of DMN are self-referential processes and episodic memory (Andrewshanna et al., 2010), while the ECN is responsible planning, decision-making, goal-directed behavior, and cognitive control (Littow et al., 2015). Previous studies in patients with OCD also reported decreased iFC between SN and DMN (Beucke et al., 2014; Posner et al., 2014; Gürsel et al., 2018), and between SN and ECN (Harrison et al., 2009; Gürsel et al., 2018). Decreased iFC between SN and DMN, as well as between SN and ECN may imply the existence of a chaotic relationship between the internal and external environment in patients with OCD, because the basic modulation function of the SN switching between DMN and ECN may decline (Fan et al., 2017a). Consequently, abnormal inter-SN iFC may be associated with patients’ difficulty in disengaging from internally self-referential thoughts, and the ability to plan goal-directed behavior to adapt the changing external environment, which may lead to the cognitive and behavioral disturbances simultaneously in OCD. In addition, reduced SN-DMN connectivity may contribute to decreased sustained attention (Posner et al., 2016) and may also be related with poor insight in patients with OCD (Fan et al., 2017b).

However, contrary to our results, greater iFC within the SN (Fan et al., 2017a,b), and increased iFC between SN and DMN, between SN and ECN were revealed by previous studies (Posner et al., 2016; Fan et al., 2017a; Wang et al., 2018). The decreased reproducibility of neuroimaging findings may be due to the intrinsically low statistical power of relatively small sample size (Button et al., 2013). Moreover, compared with previous studies, the patients with OCD in our study may have different clinical OCD subtypes (i.e., good insight and poor insight), different clinical OCD subtypes are thought to have different pathophysiology (van den Heuvel et al., 2009). Most importantly, the majority of the results from previous studies didn’t survive at the strict AlphaSim correction level of *p* < 0.001 (Fan et al., 2017a,b). Lower statistical power may induce some false-positive results in neuroimaging study (Eklund et al., 2016). Therefore, relatively large and homogeneous samples of patients with OCD and strict statistical level are needed for future studies.

In this study, we utilized the subregions of SN and found decreased intra- and inter- iFC of the SN. However, previous studies used different seed definition of SN and revealed different iFC patterns within and between SN and other network. For example, Fan et al. (2017b) used the bilateral anterior insular and dACC as SN and found increased iFC within the SN in patients with OCD; Posner et al. (2016) defined the bilateral anterior insular as SN and revealed no significant differences in iFC within the SN, but increased iFC between the SN and the DMN in patients with OCD; Stern et al. (2012) used bilateral dorsal anterior insula as SN, and discovered decreased iFC between SN and DMN in patients with OCD. The inconsistence of previous studies may attributed to assumptive and different seed definitions of the SN, which may limit to explore the iFC patterns of brain network (Stern et al., 2012; Posner et al., 2016).

Inconsistent with our hypothesis, we didn’t found any correlations between altered iFC strength and clinical symptoms in patient with OCD. We infer that the altered iFC strength within the SN subregions may be a trait change for OCD independent of the clinical variables (Guo et al., 2014), and should be investigated in future studies.

This study has several limitations. First, the relationship between the DMN and the ECN was not explored in patients with OCD. Second, different clinical OCD subtypes, such as good insight and poor insight, may have different intra- and inter- iFC at the SN in patients with OCD. Third, cognitive and behavioral information of patients with OCD were not collected in our study. Lastly, some patients with OCD had history of psychotropic medication, which may already caused changes in brain function and structure. Therefore, the patients enrolled in this study were not all drug-naïve, and the results of our study should be interpreted with caution. Future study needs to take these into consideration.

Taken together, the present study conducted a detailed investigation of SN in patients with OCD by testing for abnormalities in all subregions of the SN. Our results not only demonstrated decreased connectivity within the SN, but also reduced inter-network connectivity with DMN and ECN. Therefore, the present findings suggest that patients with OCD exhibit unique changes of iFC in SN, and provide new insight into the dysfunctional brain organization of the SN in OCD. The “triple-network” model may contribute to the clinical phenotype of OCD.

## Author Contributions

Y-HC, DL, Y-HW, XM, QH, C-CL, and L-TZ performed the experiments. Y-HC, DL, and G-DZ analyzed the data. S-FL, X-PC, and X-PW revised the manuscript. PL and Y-HC designed and conceived the experiments and wrote the manuscript.

## Conflict of Interest Statement

The authors declare that the research was conducted in the absence of any commercial or financial relationships that could be construed as a potential conflict of interest.

## Footnotes

1 http://findlab.stanford.edu/functional_ROIs.html
