# Supplementary material for: Decreased Intrinsic Functional Connectivity of the Salience Network in Drug-Naïve Patients With Obsessive-Compulsive Disorder
Source: Front Neurosci. 2018 Nov 28;12:889. doi: 10.3389/fnins.2018.00889 (PMC6279930; doi:10.3389/fnins.2018.00889)
Supplement: Supplementary file 1 [file Data_Sheet_1.doc]

**Supplementary Material**

This SN atlas used in present study was developed by the Functional Imaging in Neuropsychiatric Disorders (FIND) lab at Stanford University (Table S1 and Figure S1).

**Table S1.**

The 19 salience network subregions.

| **SN** | **Brain Region** | **Centre MNI coordinates** | | | **Voxels** |
| --- | --- | --- | --- | --- | --- |
|  |  | **x** | **y** | **z** |  |
| Anterior 1 | L MFG | -29 | 47 | 20 | 651 |
| Anterior 2 | L insula | -43 | 14 | -5 | 305 |
| Anterior 3 | L&R dACC | ±7 | 32 | 22 | 2877 |
| Anterior 4 | R MFG | 28 | 47 | 21 | 470 |
| Anterior 5 | R insula | 43 | 14 | -5 | 319 |
| Anterior 6 | L cerebellum_Crus1 | -33 | -58 | -33 | 95 |
| Anterior 7 | R cerebellum_Crus1 | 33 | -58 | -33 | 139 |
| post 1 | L MFG | -38 | 36 | 29 | 93 |
| Post 2 | L [supramarginal gyrus](../../../../../Administrator/AppData/Local/youdao/dict/Application/7.5.2.0/resultui/dict/result.html) | -58 | -38 | 36 | 1205 |
| Post 3 | L precuneus | -8 | -53 | 61 | 98 |
| Post 4 | R MCC | 13 | -30 | 42 | 56 |
| Post 5 | R SPL | 16 | -55 | 66 | 133 |
| Post 6 | R [supramarginal gyrus](../../../../../Administrator/AppData/Local/youdao/dict/Application/7.5.2.0/resultui/dict/result.html) | 58 | -38 | 36 | 1002 |
| Post 7 | L thalamus | -10 | -19 | 6 | 142 |
| Post 8 | L cerebellum_6 | -32 | -43 | -37 | 102 |
| Post 9 | L insula | -37 | -14 | -3 | 114 |
| Post 10 | R thalamus | 13 | -15 | 7 | 63 |
| Post 11 | R cerebellum_6 | 33 | -43 | -39 | 13 |
| Post 12 | R insula | 40 | -7 | -9 | 134 |

SN, salience network; MNI, Montreal Neurological Institute; MFG, middle frontal gyrus; dACC, dorsal anterior cingulated cortex; MCC, middle cingulate cortex; SPL, superior parietal lobe; L, left; R, right.


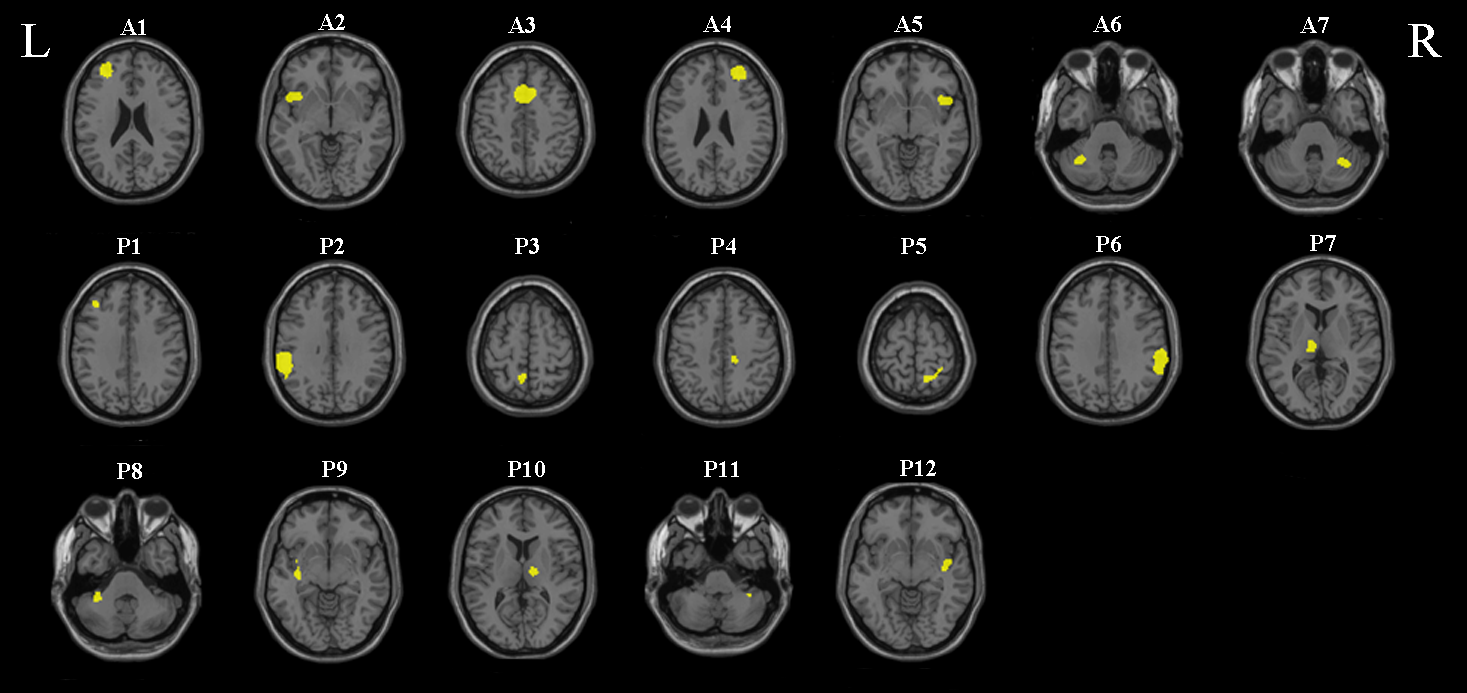


**Supplementary figure S1.** The 19 salience network subregions. L, left side; R, right side. A1, left middle frontal gyrus; A2, left insula; A3, left and right dorsal anterior cingulated cortex; A4, right middle frontal gyrus; A5, right insula; A6, left cerebellum_Crus1; A7, right cerebellum_Crus1; P1, left middle frontal gyrus; P2, left [supramarginal gyrus](../../../../../Administrator/AppData/Local/youdao/dict/Application/7.5.2.0/resultui/dict/result.html); P3, left precuneus; P4, right middle cingulate cortex; P5, right superior parietal lobe; P6, right [supramarginal gyrus](../../../../../Administrator/AppData/Local/youdao/dict/Application/7.5.2.0/resultui/dict/result.html); P7, left thalamus; P8, left cerebellum_6; P9, left insula; P10, right thalamus; P11, right cerebellum_6; P12, right insula.


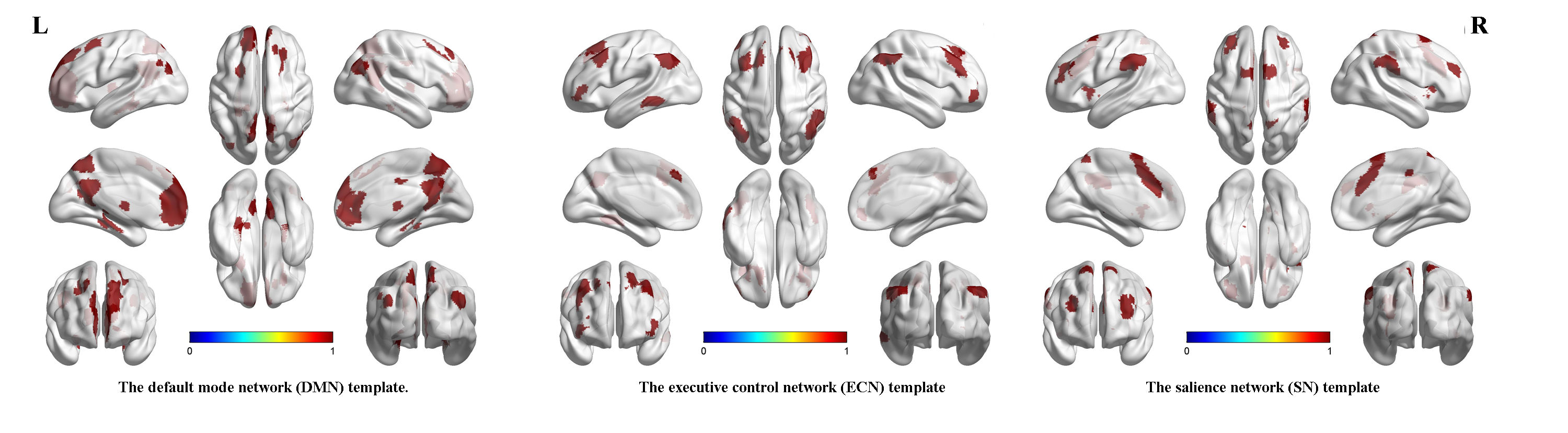


**Supplementary figure S2.** The default mode network (DMN), executive control network (ECN), and salience network (SN) template.
